# The Neurological Wake-up Test—A Role in Neurocritical Care Monitoring of Traumatic Brain Injury Patients?

**DOI:** 10.3389/fneur.2017.00540

**Published:** 2017-10-17

**Authors:** Niklas Marklund

**Affiliations:** ^1^Department of Clinical Sciences Lund, Neurosurgery, Lund University, Skane University Hospital, Lund, Sweden; ^2^Department of Neuroscience, Neurosurgery, Uppsala University, Uppsala, Sweden

**Keywords:** traumatic brain injury, neurocritial care, wake-up test, monitoring, stress response

## Abstract

The most fundamental clinical monitoring tool in traumatic brain injury (TBI) patients is the repeated clinical examination. In the severe TBI patient treated by continuous sedation in a neurocritical care (NCC) unit, sedation interruption is required to enable a clinical evaluation (named the neurological wake-up test; NWT) assessing the level of consciousness, pupillary diameter and reactivity to light, and presence of focal neurological deficits. There is a basic conflict regarding the NWT in the NCC setting; can the clinical information obtained by the NWT justify the risk of inducing a stress response in a severe TBI patient? Furthermore, in the presence of advanced multimodal monitoring and neuroimaging, is the NWT necessary to identify important clinical alterations? In studies of severe TBI patients, the NWT was consistently shown to induce a stress reaction including brief increases in intracranial pressure (ICP) and changes in cerebral perfusion pressure (CPP). However, it has not been established whether these short-lived ICP and CPP changes are detrimental to the injured brain. Daily interruption of sedation is associated with a reduced ventilator time, shorter hospital stay and reduced mortality in many studies of general intensive care unit patients, although such clinical benefits have not been firmly established in TBI. To date, there is no consensus on the use of the NWT among NCC units and systematic studies are scarce. Thus, additional studies evaluating the role of the NWT in clinical decision-making are needed. Multimodal NCC monitoring may be an adjunct in assessing in which TBI patients the NWT can be safely performed. At present, the NWT remains the golden standard for clinical monitoring and detection of neurological changes in NCC and could be considered in TBI patients with stable baseline ICP and CPP readings. The focus of the present review is an overview of the existing literature on the role of the NWT as a clinical monitoring tool for severe TBI patients.

## Introduction

Intense clinical monitoring is an integral part of the management or traumatic brain injury (TBI) patients. Neurological worsening, commonly defined as a decrease of two or more points on the motor component of the Glasgow Coma Scale (GCS-M) score, may occur rapidly and is associated with a poor outcome in TBI. Clinical deterioration may be caused by, e.g., ongoing hemorrhage or increased brain swelling (1, 2) stressing the importance of repeated clinical evaluations. In addition, information from these neurological examinations may prove important in clinical decision-making, and lead to improved patient outcome. A swift neurological examination in the emergency setting can assess the gross structural integrity of the nervous system, enable an assessment of the injury severity and provide a prognostication tool for TBI. The components of the neurological examination used for monitoring can vary depending on the clinical situation, although assessment of the level of consciousness, neurological motor function, and assessment of pupillary size and reactivity is a minimal requirement.

The importance of repeated neurological evaluations was highlighted in the 1970s, when a large number of TBI patients were identified who on admission to the hospital were awake and able to talk but later died. The entity “talk-and-die” was coined (3), describing individuals in whom the severity of the initial, primary injury was insufficient to explain the poor outcome and that the occurrence of secondary, and presumably preventable and “avoidable,” factors resulted in the fatal exacerbation of the disease. These findings prompted increased awareness and improved organization of TBI care, aided by the standardization of the neurological assessment through the introduction of the Glasgow Coma Scale (GCS) score in 1974 (4). Since TBI is a markedly heterogeneous disease that commonly shows an unpredictable and dynamic clinical course, adherence to protocols for neurological surveillance including repeated clinical evaluations of TBI patients is mandatory. Stricter guidelines and management protocols have presumably contributed to gradually reduced case fatality rates (5, 6), acknowledging that virtually all forms of TBI carry an inherent risk of exacerbation over time. The risk factors for neurological worsening in the mild–moderate TBI population have been addressed in numerous previous publications and guidelines (7–10), aiding the emergency room physician in the often difficult decision whether to perform neuroradiological investigations, admit for clinical monitoring or discharge the patient.

The use of prehospital sedation, paralysis, and intubation frequently used at the scene of the accident (11, 12) makes an assessment of neurological status of the TBI patient difficult. Following initial resuscitation of severe TBI patients, a neurological examination to obtain a post-resuscitation GCS score is recommended (13, 14) for TBI severity grading and for clinical decisions (15). Most severe TBI patients, after radiological investigations and surgical evacuation of space-occupying mass lesions, will also require continued care in a neurocritical care (NCC) unit. The NCC foundations for managing severe TBI consist of controlled ventilation, stress reduction using, e.g., continuous sedation, neuroimaging, and multimodality monitoring including the measurement of intracranial pressure (ICP) and cerebral perfusion pressure (CPP). Importantly, up to 40% of TBI patients show a clinically relevant neurological worsening within the first 48 NCC hours (16–18), arguing for repeated neurological examinations also during NCC.

Clinical examinations in NCC for severe TBI are controversial since they pose a dilemma—while sedation interruption is needed for the important neurological evaluation, an undesired stress response is commonly elicited. Is clinical monitoring using neurological assessments justified in modern NCC, and does the obtained information of the evaluation outweigh its potential risks? Furthermore, in the era of multimodality monitoring, what additional information is provided by neurological examinations and do they lead to changed management of the severe TBI patients? In the present overview, the rather scarce literature on neurological evaluation [here named the neurological wake-up test, NWT (19)], used as a clinical monitoring tool for severe TBI patients is discussed. Although the term “wake-up test” is used, it should be remembered that the response to interruption of sedation should not be regarded as an “awakening”; instead the response may be more comparable to an arousal reaction (20). Several terms to describe interruption of sedation strategies during NCC and/or the general intensive care units (ICUs) are used interchangeably in the medical literature, and may include protocols with our without concomitant evaluation of the neurological status. Conversely, sedation interruption or sedation lightening allowing for spontaneous breathing with the aim of speeding ventilator weaning is used in some protocols without simultaneously performing an NWT. Spontaneous awakening trials (SATs), spontaneous breathing trials (SBTs), daily interruption of sedation (DIS or IS-) trials, and lightening of sedation are examples of the used terminology. For the purpose of this review, medical databases (Medline, Scopus, and PsychINFO) were searched using the terms TBI, or any combination of brain or head trauma/injury, together with wake-up test, SBT, SAT, and/or lightening/interruption of sedation.

## Continuous Sedation and Sedation Interruption in NCC and General Intensive Care

Continuous sedation is used in general ICUs to prevent pain and anxiety, control agitation, and minimize patient discomfort, as well as to enable endotracheal tube tolerance needed for controlled mechanical ventilation (21–23). Additional NCC-specific aims of continuous sedation include the prevention of stress-related secondary insults, the reduction of cerebral energy metabolism and oxygen consumption, and seizure, ICP and temperature control (24). There are several sedatives for use in the NCC, the choice and combination of which may influence ICP and CPP control as well as cardiovascular stability. The most commonly used sedatives in NCC are arguably propofol and midazolam although compounds such as the selective α2-adrenergic agonist dexmedetomidine (25, 26) or the *N*-methyl-d-aspartate receptor antagonist ketamine (23) are more recent additions to the sedation armamentarium. The selected sedative, due to its plasma half-life and/or potential for lingering central nervous system effects, obviously influences the possibility of using the NWT in NCC monitoring.

Thus, continuous sedation is an integral part of both general ICU and NCC treatment protocols. However, this strategy is not without adverse effects since excessive doses of sedatives may lead to significant morbidity (27–29). Continuous sedation was also repeatedly shown to increase the incidence of ventilator-associated pneumonias, prolong mechanical ventilation, and result in a higher mortality in ICUs (27, 29–31). Since delayed weaning from mechanical ventilation increases the risk of infections, it is desirable to reduced ventilator time (30, 32). These observations led to the implementation of daily interruption of continuous sedation (DIS) trials in general ICU (27, 29–31). A DIS protocol in combination with SBTs reduced the duration of mechanical ventilation and length of stay in general ICU, without increasing the complication frequency (33) or impairing long-term cognitive, psychological, and functional outcomes (27, 31, 34, 35). These data implied that DIS is beneficial in general ICU, although some uncertainty of patient safety and/or agitation has persisted and this strategy is used in only ca. 30–40% of ICU patients (27). However, in a study of Australian ICU patients, sedation interruption guidelines was not associated with a reduced duration of mechanical ventilation, and similar results were observed when a protocol-driven weaning protocol was evaluated (36). In a meta-analysis evaluating 699 critically ill ICU patients, DIS protocols were not found to reduce ventilator-associated pneumonias, duration of mechanical ventilation, length of ICU stay, or mortality although it did reduce the risk of tracheostomy (37). Finally, no strong evidence that DIS alters the duration of mechanical ventilation, mortality, length of ICU or hospital stay, adverse event rates, drug consumption, or quality of life was provided in a recent Cochrane review (32). In fact, an analgesia-delirium-sedation protocol, using carefully titrated sedation aimed to limit sedation depth and duration, was effective in reducing ventilator days and hospital stay in ICU patients without using DIS (38).

The current literature does not convey a clear message or substantial proof for benefit of minimizing sedation although the negative consequences of over-administration of sedatives are well established. Instead, protocol-driven control of sedation in combination with sedation scales and the use of sedatives with a short half-life may be equally effective to DIS for ICU patients (20). The level of evidence for minimizing complications by DIS in NCC is even lower. In a randomized control trial, a subgroup of TBI patients did not show significantly decreased ventilator time or ICU stay compared to controls when sedation was interrupted on a daily basis [see Table 1; (37)]. In contrast, the ability of sedation to reduce cerebral metabolic demand, ICP control, temperature management, and seizure control in NCC are undisputed.

**Table 1:** Summary of published articles on the neurological wake-up test (NWT) in traumatic brain injury (TBI) and their key findings.

Reference	TBI patients (*n*)	Sedative(s)	Key outcome measure	Main conclusion
(19)	12 (+9 SAH)	Propofol	ICP increased with 69% and CPP by 5% during the NWT. MABP and pulse rate increased Peripheral oxygen saturation unchanged.	NWT increased ICP and MABP
(39)	38 TBI (21 TBI and NWT, 17 TBI controls)	Mainly propofol and remifantanil	Length of stay and days on mechanical ventilation not significantly altered	No ICU benefit of the NWT
(40)	17	Propofol	ICP and CPP increased Interstitial levels of glucose, lactate, pyruvate, glutamate, glycerol, and the lactate/pyruvate ratio unchanged measured by microdialysis. SjvO_2_ and PbtiO_2_ unchanged	No evidence of an exacerbated brain injury by the NWT
(41)	24	Propofol	ICP and CPP increased Epinephrine, norepinephrine, and ACTH levels in blood increased Cortisol in saliva increased Modest absolute increases of stress hormone levels	NWT induced a biochemical stress response
(42)	TBI *n* = 4SAH *n* = 14; ICH *n* = 2	Combination of DEX, midazolam, propofol and fentanyl	54 NWTs were attempted, 1/3 stopped due to increased ICP. PbtiO_2_ decreased in NWT failures. In only one NWT was neuroworsening detected. ICP and MABP increased	Many NWTs stopped for safety concerns, no benefit of the test
(43)	242; NWT performed in 96 patients	Propofol	Early, <24 h, NWT stopped in 40% of patients (*n* = 27) Reasons for NWT failure was “neurological” in 71% (increased ICP or status epilepticus in 33% of these) or respiratory in 26%	NWT failure associated with subdural hematoma thickness or GCS score <5

*ICP, intracranial pressure; CPP, cerebral perfusion pressure; SjvO_2_, jugular venous saturation; PbtiO_2_, brain tissue oxygenation; MABP, mean arterial blood pressure; DEX, dexmedetomidine; ICU, intensive care unit; ACTH, adrenocorticotrophic hormone; ICH, intracerebral hemorrhage; SAH, subarachnoid hemorrhage; GCS, Glasgow Coma Scale*.

At present, many controversies remain with regard to interruption of sedation in TBI patients where continuous sedation is part of the treatment strategy. At the current level of evidence, potential systemic benefits of the procedure derived from general ICU care may not be used as a key argument in favor of using NWTs in TBI care.

## Indications for the NWT in NCC

Since there is no clear-cut evidence for a clinical benefit of sedation interruption in TBI patients, what other possible indications for the NWT are there? In particular, what additional information is sought by the NWT in the sedated and monitored TBI patient? The NWT is not mentioned in available TBI guidelines (13), and the use of the NWT may vary considerably among NCC centers. In our own Scandinavian survey, ca. 50% of NCC centers never used the NWT in daily routine care of TBI patients and there was a marked variation in the frequency of the NWTs in the remaining ones (44). Compared to midazolam, propofol sedation may facilitate use of the NWT due to its shorter half-life and one factor explaining the variable use of the NWT in NCC may be the choice of sedatives in certain centers (44).

Advocates for using the NWT in TBI argue that this test is the only monitoring tool that can reliably detect clinically important neurological improvement or deterioration, including the emergence/exacerbation of focal neurological deficits (45). Clinical changes detected by the NWT could include signs of progressive brainstem involvement or provide clinical evidence for successful surgery of intracranial mass lesions. In addition, based on information obtained by the NWT clinical management may be more aggressive in deteriorating patients, or lead to, e.g., extubation in those showing signs of recovery (16).

The information obtained by the NWT may also facilitate clinical decisions on, e.g., changing ventilator strategies, surgical treatment, or the ordering of neuroradiological investigations. Thus, the indications for the NWT are obvious. There are however numerous other neuromonitoring possibilities in modern NCC that in addition to ICP and CPP monitoring include brain neurochemistry [intracerebral microdialysis (MD)], brain tissue oxygen monitoring (PbtiO_2_), and jugular venous oxygen saturation (SjvO_2_) monitoring, among others. These additional tools help to control and maintain intracranial dynamics with the aim to prevent, detect, and treat secondary insults known to exacerbate the primary injury (46, 47). One caveat of neuromonitoring is that although ICP elevations and brain herniation are commonly linked, they can occur independently (48). This means that in, e.g., temporal contusions or following decompressive craniectomy, worsening of the intracranial situation detectable by the NWT may occur without distinctly increased ICP. Since continuous sedation will mask clinical exacerbation, the NWT remains a golden standard for the detection of neurological deterioration even in the presence of advanced neuromonitoring (49).

## The NWT-Technical Aspects and Contraindications

**Figure 1:**
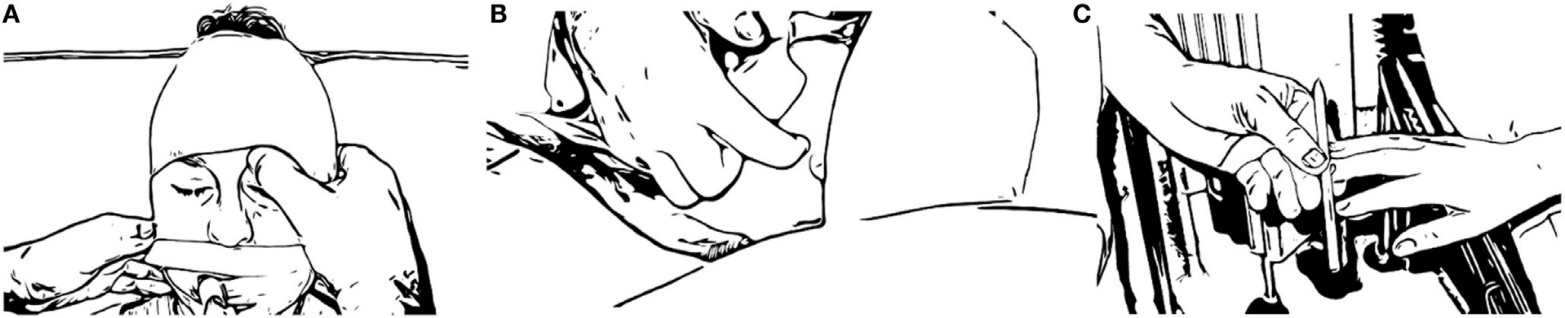
Illustration on how to evaluate the pain response, an integral component of the NWT of unconscious patients unable to obey commands. Apply a steady pressure at the medial aspect of the periorbital rim, at the supraorbital notch **(A)**, or preferably at the angle of the jaw **(B)**. After the pain response has been noted, a peripheral pain stimulus is provided by compressing the fingertips with a pencil **(C)** and, e.g., a localization, withdrawal, flexion, or extension response can be recorded (52). Based on the response of the patient, the motor component of the Glasgow Coma Scale can then be evaluated. The pupillary response to light and the presence of anisocoria as well as any focal neurological deficits are also noted.

For the NWT to be considered, it is imperative that the patient shows stable ICP and CPP values as well as PbtiO_2_ values at baseline, during continuous sedation. Conversely, the NWT should not be used in patients with ICP and/or CPP problems, or in patients with marked hyperthermia, status epilepticus, and/or barbiturate treatment. A prerequisite for the NWT is obviously that the TBI patient is without sedation at the time of the neurological assessment. Thus, when an NWT is planned, the continuous infusion of sedatives is interrupted although a low dose of analgesics during the wake-up test may preferably be maintained (50). To perform the NWT (Figure 1), the patient should be placed in the supine position. The time from interruption of sedation to the NWT may be highly variable (19), and careful monitoring of ICP and CPP during this time is needed. Prior to performing the NWT, the patient should be evaluated to ensure that he/she is sufficiently awakened from the sedation to enable further assessment. Then, always with a watchful eye on the ICP/CPP readings, the patient is requested to obey simple commands (squeeze a hand, move a foot, etc.) and the evaluator scores the response according to the GCS-M. If the patient does not obey commands even after repeated testing, a painful stimulus at the angle of the jaw is delivered and the best GCS-M response (e.g., withdrawal, stereotypic flexion/extension, localization; Figure 1) is noted. Neuroworsening, e.g., deterioration of the level of consciousness defined as a drop in the GCS-M score of ≥two points (51), mandates further investigations. In addition, each extremity should be assessed for the presence of focal neurological deficits and the pupil diameter, presence of anisocoria and the direct and indirect pupillary light reflexes be evaluated (Figure 1).

## Studies of the NWT in Severe TBI

To date, there are only scarce reports evaluating the NWT in NCC (Table 1) and there are no clinical guidelines for using, or avoiding, the NWT. In the only randomized control trial addressing a daily interruption of continuous sedation (DIS) protocol in a small subgroup of TBI patients, significantly decreased days on mechanical ventilation or length of stay in the NCC were not observed (39). However, these results were obtained from only 21 TBI patients who were compared to 17 TBI controls in whom continuous sedation was used. Yet, the duration of mechanical ventilation was 7.7 days in TBI patients receiving the DIS protocol vs. 11.6 days in the controls, and the length of NCC stay was 14 vs. 17 days, respectively.

The basic idea of an NWT is to evaluate and detect alterations in the neurological status of TBI patients. The potential benefits or risks associated with the procedure have only been studied in a few reports, most of which are from our own group. In an initial report, 127 NWTs in 12 TBI and 9 subarachnoid hemorrhage (SAH) patients were evaluated (19). In all NWTs, a stress response was observed including transient increases in pulse rate and increased mean arterial blood pressure (MABP). The duration from the interruption of sedation until the NWT could be performed was variable, with a mean of 23 min and a maximal duration of 109 min. The ICP increased by a mean of 69%, from 13 to 23 mmHg, in TBI patients while the CPP showed a non-significant 5% increase during NWT (19). In 9 of the TBI patients, the ICP levels reached >30 mmHg where the highest recorded ICP level was 71 mmHg during the NWT. In two TBI patients, the CPP increased to >130 mmHg. In addition, the CPP levels decreased to <50 mmHg in four TBI patients with the lowest recorded CPP being 29 mmHg. These ICP increases and/or CPP changes were predominately brief and transient, and it was concluded that the NWT procedure was safe in a majority of TBI patients. As mentioned, the ICP and/or CPP changes were marked in a subset of patients where an additional insult to the injured brain cannot be excluded. In addition, even short-lived ICP increases may be associated with a worse outcome in TBI (19, 53).

To address the concern that a secondary insult to the injured brain could be induced by the NWT, 17 patients with severe TBI (11 focal TBI, 6 diffuse/mixed TBI) were studied (40). The effects of the NWT on PbtiO_2_, SjvO_2_, and arterial-venous differences (AVDs) of O_2_, glucose, and lactate, and interstitial neurochemistry as measured by cerebral MD were evaluated. The PbtiO_2_ was analyzed in 51 NWTs of 8 TBI patients and remained unaltered and stable throughout the NWT procedure. At baseline, two patients had a PbtiO_2_ < 10 mm Hg, one of which showed increasing PbtiO_2_ levels during the NWT. Similarly, the SjvO_2_ and AVDs were analyzed in six TBI patients for a total of 28 NWTs. No jugular venous catheter readings were exacerbated by the NWT. One patient had a jugular venous saturation <50% on two occasions at baseline, which increased to >60% during both NWTs. Finally, MD was used in 12 TBI patients using the regular perfusion flow rate of 0.3 µL/min in 21 NWTs or, in order to better appreciate any rapid changes potentially induced by the NWT, an increased flow rate of 1.0 μL/min in 28 NWTs. Regardless of the perfusion flow rate, the NWT did not alter interstitial glucose, lactate, glycerol, glutamate, or the lactate/pyruvate ratio. In this and previous reports, the ICP and CPP levels, MABP, and pulse rate were significantly increased by the NWT (19, 40, 41). However, the results of MD, SjvO_2_, and PbtiO_2_ monitoring suggested that despite an NWT-induced stress response, no evidence of an additional brain injury was observed (40).

Severe TBI is *per se* accompanied by a systemic biochemical stress response including the release of stress-related hormones such as cortisol and the catecholamines norepinephrine and epinephrine (54–57). Arguably, continuous sedation attenuates this stress response, which aids in controlling ICP. As previously mentioned, one potential risk of using the NWT is the exacerbation of the TBI-induced stress response. NWT-induced changes in plasma adrenocorticotrophic hormone (ACTH), as well as serum norepinephrine and epinephrine levels were evaluated and compared to baseline samples drawn during continuous sedation and prior to NWT. In addition, saliva cortisol was collected by a sublingual swab (41). In 8 patients and 12 NWTs, the catecholamines epinephrine and norepinephrine levels increased by 87.5 and 40.4% from baseline, respectively. For ACTH and cortisol, the NWT-induced increases were 72.5 and 30.7%, respectively. There was no association between the increased levels of these stress hormones and peak ICP or the level of consciousness. Although the NWT significantly increased all stress hormone levels when compared to baseline, their increases in absolute numbers were minor. This study provides an additional argument that the NWT causes a stress response, which however is mild in the majority of TBI patients.

Finally, in a mixed cohort of brain-injured patients of which only four had a severe TBI, interruption of sedation was avoided in 47% of eligible patients due to critical ICP levels, hemodynamic instability, and a need for sedation (42). The authors then performed 54 NWTs, in their article named interruption of sedation trials. Of these, a third of trials could not be completed due to ICP crisis, agitation, desaturation, or a combination of these factors. In addition, reduced PbtO_2_ levels were commonly observed. In only one completed trial was a neuroworsening detected and it was argued that monitoring with MD enabled the detection of this deterioration prior to the NWT. Although there were only few TBI patients in this study, the results emphasize that not every TBI patient should be subjected to an NWT and that careful risk stratifications and individualized assessments are needed (20, 42, 58). The results of this study were also supported by a retrospective report on the use of the NWT in severe TBI in which failure to complete the test was common (43).

## Pros and Cons of the NWT as a Monitoring Tool for Severe TBI

Since one key aim of NCC for TBI is to avoid secondary insults, does the obtained information by the NWT justify the risk of increased ICP and/or decreased CPP? How detrimental is the stress response, observed in virtually all NWT studies, and are there any identifiable clinical benefits of the test (Table 2)? Interruption of continuous sedation has not been shown to cause long-term psychiatric problems such as posttraumatic stress disorder or recall of event (31). In experienced hands, the NWT-induced stress response is mild in the majority of patients (19), at least in those TBI patients who are stable at baseline, and adverse effects such as self-extubation are rare. Thus, when the ICP, CPP, and/or pBTiO_2_ recordings assessed prior to sedation interruption are within accepted limits, the NWT could be considered.

**Table 2:** Summary of some important pros and cons on the neurological wake-up test in traumatic brain injury.

Pro	Con
Detection of changes in neurological status leads to more active management	Induces a stress response with increased ICP, changes in CPP, hypertension
Reduced risk for ventilator-associated pneumonias, reduced ICU stay and less time on ventilator?	No clinical benefit over multimodality monitoring
An important clinical decision tool	Increases brain metabolism and oxygen consumption

*ICU, intensive care unit; ICP, intracranial pressure; CPP, cerebral perfusion pressure*.

There is a complete lack of Level I evidence for using, or refraining from using, the NWT in NCC and its use may predominately be based on personal preferences and/or experience as well as locally adopted guidelines and traditions. In the previous study by Helbok and colleagues, evidence of a new focal neurologic deficit was found only in one SAH patient and in no TBI patient, although in this study only four TBI patients were included (42). Surprisingly, systematic analysis of the information achieved by the NWT in TBI and what clinical decisions are made based on this information is rare. Such studies should be feasible to design and be crucial in interpreting the role for NWTs in TBI management. If the NWT does not provide information needed for important clinical decisions, its use cannot be justified. Conversely, if the NWT leads to more active management, detection of relevant causes for neuroworsening and/or improvement, and guides clinical decisions then the NWT-induced stress response can be motivated if the patient is carefully monitored during the procedure.

There are also many arguments against the NWT. As stated in the article by Helbok and colleagues, the NWT may only rarely add clinical information of importance over other monitoring tools (42). In addition, the NWT-induced stress response is likely to increase cerebral metabolism and oxygen consumption, factors not desirable in the vulnerable TBI patient (20). A valid argument against the routine implementation of the test is also the exclusion of patients unstable at baseline, since these individuals may be those in whom the NWT would add the most useful information. Although sedation *per se* has never been shown to positively influence outcome, it is clearly a treatment in itself for ICP and CPP control, stress reduction, and attenuation of cerebral energy metabolism (24, 50, 59).

Due to the lack of solid data, the central question remains—how often can the NWT detect an altered neurological condition that will influence patient management? This question calls for additional studies. If no clinical benefits can be identified from the NWT, there are other available sedation algorithms in combination with sedation scales that may reduce the risk of over-sedation (42). Recently, it was suggested that in all patients at risk for ICP elevations, in those undergoing active temperature lowering therapies and in those treated for refractory status epilepticus the NWT should be avoided. If these factors are not present, NWTs/interruption of sedation protocols can be used as in general ICU care (23). It appears feasible that modern multimodal monitoring and NWT may co-exist, and that other monitoring tools can be used to define in what TBI patients it is safe, or unsafe, to perform an NWT.

## Conclusion

The aim of this review was to assess the available literature on the use of the NWT as a monitoring tool in the NCC management of TBI patients. To date, there are no strong arguments for a clinical benefit of the NWT in severe TBI patients. An obvious goal of continuous sedation is also the reduction of cerebral energy metabolic demands in severe TBI. In the majority of evaluated studies, the NWT is associated with a variable systemic stress response. However, there are no data clearly showing that this stress response results in a significant secondary brain injury. Patients with unstable ICP and/or CPP levels, hyperthermia and/or status epilepticus should not be subjected to the test. In others, when used by personnel experienced in the interruption of sedation required for the NWT, the test may in medically stable TBI patients provide useful clinical information such as neuroworsening or neuro-improvement and be used in daily clinical decision-making. There is thus an argument for implementing the NWT in management protocols for selected TBI patients. In summary,

– From a scientific perspective, there is neither evidence against the use of the NWT nor in its favor.
– The NWT is associated with a stress response, the consequences of which have not been fully elucidated. To date, there is no clear evidence for a secondary brain injury induced by the NWT.
– Factors such as local management traditions, experience of the nursing staff and/or the choice of sedatives appears to decide the use and frequency of the NWT.
– An individualized assessment based on neuromonitoring and neuroimaging parameters is needed to decide in which patient the NWT is safe.
– The choice is not between multimodality monitoring and the NWT; TBI management strategies may well include a combination of both.
– A study systematically evaluating the clinical decision-making based on information obtained by an NWT appears feasible and could enhance the knowledge of the pros and cons as well as define the role of the NWT in modern-day NCC.

## Author Contributions

The author confirms being the sole contributor of this work and approved it for publication.

## Conflict of Interest Statement

The author declares that the research was conducted in the absence of any commercial or financial relationships that could be construed as a potential conflict of interest.
